# Cannabinoid Drugs-Related Neuroprotection as a Potential Therapeutic Tool Against Chemotherapy-Induced Cognitive Impairment

**DOI:** 10.3389/fphar.2021.734613

**Published:** 2021-11-12

**Authors:** Laura Boullon, Raquel Abalo, Álvaro Llorente-Berzal

**Affiliations:** ^1^ Pharmacology and Therapeutics, School of Medicine, National University of Ireland, Galway, Ireland; ^2^ Centre for Pain Research, National University of Ireland, Galway, Ireland; ^3^ Galway Neuroscience Centre, National University of Ireland, Galway, Ireland; ^4^ Área de Farmacología y Nutrición, Departamento de Ciencias Básicas de La Salud, Universidad Rey Juan Carlos (URJC), Alcorcón, Spain; ^5^ Unidad Asociada I+D+i Del Instituto de Química Médica (IQM), Consejo Superior de Investigaciones Científicas (CSIC), Madrid, Spain; ^6^ High Performance Research Group in Physiopathology and Pharmacology of the Digestive System NeuGut-URJC, Madrid, Spain; ^7^ Working Group of Basic Sciences in Pain and Analgesia of the Spanish Pain Society (Grupo de Trabajo de Ciencias Básicas en Dolor y Analgesia de La Sociedad Española Del Dolor), Madrid, Spain

**Keywords:** chemotherapy-induced cognitive impairment, cannabinoid drugs, endocannabinoid system, neuroinflammation, oxidative stress

## Abstract

In recent years, and particularly associated with the increase of cancer patients’ life expectancy, the occurrence of cancer treatment sequelae, including cognitive impairments, has received considerable attention. Chemotherapy-induced cognitive impairments (CICI) can be observed not only during pharmacological treatment of the disease but also long after cessation of this therapy. The lack of effective tools for its diagnosis together with the limited treatments currently available for alleviation of the side-effects induced by chemotherapeutic agents, demonstrates the need of a better understanding of the mechanisms underlying the pathology. This review focuses on the comprehensive appraisal of two main processes associated with the development of CICI: neuroinflammation and oxidative stress, and proposes the endogenous cannabinoid system (ECS) as a new therapeutic target against CICI. The neuroprotective role of the ECS, well described in other cognitive-related neuropathologies, seems to be able to reduce the activation of pro-inflammatory cytokines involved in the neuroinflammatory supraspinal processes underlying CICI. This review also provides evidence supporting the role of cannabinoid-based drugs in the modulation of oxidative stress processes that underpin cognitive impairments, and warrant the investigation of endocannabinoid components, still unknown, that may mediate the molecular mechanism behind this neuroprotective activity. Finally, this review points forward the urgent need of research focused on the understanding of CICI and the investigation of new therapeutic targets.

## Introduction

The occurrence of sequelae after chemotherapeutic treatment has recently attracted increasing interest, particularly given the higher life expectancy of those with a lived experience of cancer. The cognitive alterations described following cancer experience normally occur during pharmacological treatment of the disease, however, it can prevail long after the cessation of therapy. This phenomenon is known as chemotherapy-induced cognitive impairment (CICI), chemofog or chemobrain. Preclinical research has shown that chemotherapeutic agents such as oxaliplatin, paclitaxel, cyclophosphamide, methotrexate, 5-fluorouracil or doxorubicin can induce short- and long-term deleterious effects in working memory and fear and spatial learning in a wide variety of rodent models (Table 1). Moreover, neuroimaging studies have collected data from patients following chemotherapeutic regime supporting chemotherapy induced alterations on brain structure and plasticity. These studies showed the presence of cognitive alterations independently on the tumour location; suggesting that chronic chemotherapy treatment may induce alterations on cognitive functionality (Wefel and Schagen, 2012; Conroy et al., 2013; McDonald et al., 2013; Sleurs et al., 2016).

**TABLE 1:** Summary of cognitive deficits induced by chemotherapeutic drugs in preclinical animal models of chemotherapy-induced cognitive impairment (CICI).

Chemotherapeutic drug	Animal model	Regime	Cognitive impairments observed	References
Cyclophosphamide (CPA)	Young adult male ICR mice	One i.p. administration (40 mg/kg)	• CPA induced deficits in memory retention in the PAT and the NOR 12 h after administration	Yang et al. (2010)
			• These CPA-related effects on cognition were not observed 10 days after drug administration	
	Young adult male ICR mice	Weekly i.p. administration for 4 consecutive weeks (80 mg/kg per administration)	• Learning deficiencies in the PAT.	Hou et al. (2013)
			• Impairment of spatial memory in the Y-maze	
	Young adult male athymic nude rats	Weekly i.p. administration for 5 consecutive weeks (50 mg/kg per administration)	• CPA administration caused an impairment of spatial memory in the NLR.	Christie et al. (2012)
			• In the FC paradigm, CPA caused a decrease of freezing upon re-exposure to the context, but not to the cue	
Oxaliplatin (OXA)	Male and female hooded Wistar rats	One i.p. administration (6 mg/kg)	• Male and female animals treated with OXA exhibited a deficit of working memory in the NOR.	Johnston et al. (2017)
			• OXA induced a significant impairment of spatial memory in the NLR.	
			• In the FC paradigm, OXA impaired the renewal of extinguished fear conditioning for up to 19 days after administration	
	Male Sprague-Dawley rats	One i.p. administration (12 mg/kg)	• OXA administration induced an impairment in the renewal of extinguished fear in the FC paradigm	Sharpe et al. (2012)
	Male hooded Wistar rats	Weekly i.p. administration for 3 consecutive weeks (0.6, 2 and 6 mg/kg per administration)	• Only the highest dose of OXA (6 mg/kg) induced a gradual deterioration of the recognition memory in the NOR. This impairment became appreciable 4 months after and lasted up to 11 months	Fardell et al. (2015)
			• In the NLR the lower doses of OXA (0.6 and 2 mg/kg) induced a deficit of spatial memory 15 and 30 days after treatment, although this deleterious effect was not observed 4 and 11 months after OXA administration	
			• The highest dose (6 mg/kg) induced a long lasting (up to 11 months after administration) deficit of spatial memory in the NLR.	
Cisplatin	Infant and adolescent male Sprague-Dawley rats	Weekly i.p. administration for 5 consecutive weeksd (2 mg/kg per administration)	• Cisplatin induced in infant and adolescent animals an impairment of the recognition memory in the NOR.	John et al. (2017)
			• Only adolescent animals exhibited an impairment of spatial memory in the NLR.	
			• In the FC paradigm, cisplatin impaired contextual memory, but not cued memory, of infant and adolescent animals	
5-Fluorouracil (5-FU)	Young adult male C57BL/6 J mice	One i.p. administration (75 mg/kg)	• 5-FU caused short-term (2–12 weeks) impairments of spatial memory in the NLR and the Barnes maze. Likewise, 5-FU impaired recognition memory in the NOR.	Seigers et al. (2015)
			• In the long term (15–25 weeks) only the spatial memory impairment in the NLR persisted	
Methotrexate (MTX)	Male Sprague-Dawley rats	One i.p. administration (20 mg/kg)	• Animals treated with MTX exhibited in the short-term deficits of memory retention in the PAT and an impairment of spatial memory in the Y-maze	Shalaby et al. (2019)
	Infant female C57BL/6 J mice	One i.p. administration (20 mg/kg)	• Administration of MTX during infancy induced in the adulthood an impairment of spatial memory in the Morris water maze	Elens et al. (2019)
	Young adult male Long Evans rats	- One i.t. administration (0.5 mg/kg)	• Both administration schedules of MTX induced a deficit in recognition and spatial memory measured by the NOR and the NLR respectively	Vijayanathan et al. (2011)
		- Four i.t. administrations over 10 days (0.5 mg/kg per administration)	• Repeated MTX administration induced a longer deleterious effect on cognition than the single administration protocol	
	Infant male and female Swiss-Webster mice	Daily i.p. administration for 3 consecutive days (2 mg/kg per administration)	• Infant administration of MTX induced in the adolescence an impairment of recognition memory in the NOR.	Bisen-Hersh et al. (2013)
Paclitaxel	Young adult male Sprague-Dawley rats	Four i.p. administrations every 2 days (2 mg/kg per administration)	• Impairment of spatial memory in the Morris water test	(Li et al., 2017), (Li et al., 2018)
	Young adult male C57BL/6 J mice	One i.p. administration (33 mg/kg)	• Paclitaxel induced in the short (2–12 weeks) and the long term (15–25 weeks) an impairment of spatial memory in the NLR.	Seigers et al. (2015)
Doxorubicin (DOX)	Young adult male C57BL/6 J mice	One i.v. administration (5 or 10 mg/kg)	• The lowest dose (5 mg/kg) impaired spatial memory in the NLR.	Seigers et al. (2015)
			• The highest dose (10 mg/kg) induced an impairment of the recognition memory in the NOR and the spatial memory in the NLR and the Barnes maze	
	Young adult male Wistar rats	Four i.p. administrations every 2 days (2 mg/kg per administration)	• DOX caused an impairment of spatial memory in the Morris water maze and memory retention in the PAT.	Park et al. (2018)
	Young adult male Wistar rats	One administration every 5 days over 50 days (2.5 mg/kg)	• DOX impaired recognition memory in the NOR.	Verma et al. (2017)

- Chemotherapeutic agents: 5-FU, 5-Fluorouracil; CPA, cyclophosphamide; DOX, doxorubicin; MTX, methotrexate; OXA, oxaliplatin.

- Type of administration: i.p., intraperitoneal; i.t., intrathecal; i.v., intravenous.

- Behavioural test: FC, fear conditioning; NLR, novel location recognition test; NOR, novel object recognition test; PAT, passive avoidance test.

Despite the great number of antineoplastic drugs available in the market, only a few of them have been tested on preclinical and clinical studies of CICI, emphasizing the lack of clinical evaluation of cognition-related side effects. In addition, the majority of models investigating CICI have limited their attention on non-CNS cancer types (Wefel and Schagen, 2012), especially on breast cancer, biasing thus the investigation of CICI into one sex population and type of cancer disease.

Among the most common cognitive deficiencies reported, are those of short-term working and visuospatial memories, verbal ability, executive functions and attention span (Conroy et al., 2013; Du et al., 2013; McDonald et al., 2013; Sleurs et al., 2016). These deficiencies are difficult to detect since the cognitive levels observed in CICI patients are often placed at the lower end of the normal range of the population. In addition, the lack of approved tests for CICI diagnosis complicates medical evaluation (Horowitz et al., 2018; Nguyen and Ehrlich, 2020). Similar limitations are observed in the cognitive rehabilitation of CICI patients. The current, palliative, therapies available involves physical activity and cognitive-behavioural therapy (Kesler et al., 2013; Ferguson et al., 2014; Fernandes et al., 2019). Even though these therapies seem to improve the life quality of the patients, they require a lot of time, effort and economical aids. Therefore, the ongoing investigation of CICI leads the attention to develop new pharmacotherapies attending to the neurobiological alterations associated with this disease.

There are a great number of biological mechanisms that seem to be implicated in the cognitive deficits induced by chemotherapy agents, including: direct neurotoxic effects, impaired neurogenesis or increased death of nervous cells, white matter abnormalities, inflammatory responses, oxidative stress and even alterations in the levels of sex and stress hormones (Sleurs et al., 2016; El-Agamy et al., 2019; Mounier et al., 2020; Nguyen and Ehrlich, 2020).

The endogenous cannabinoid system (ECS) is a complex signalling system comprised of cannabinoid type 1 (CB_1_) and cannabinoid type 2 (CB_2_) receptors; endocannabinoid ligands: anandamide (AEA) and 2-arachidonoylglycerol (2-AG); and catabolizing enzymes: fatty acid amide hydrolase (FAAH) and monoacylglycerol lipase (MAGL) (Viveros et al., 2012; Kaur et al., 2016; Woodhams et al., 2017; Fraguas-Sánchez et al., 2018; Gorzkiewicz and Szemraj, 2018; Micale and Drago, 2018; Zou and Kumar, 2018). Other related biogenic lipids such as oleoylethanolamine (OEA) and palmitoylethanolamine (PEA) are also included within the ECS as endocannabinoid-related compounds (Di Marzo, 2018). Interestingly, pharmacological modulation of the ECS has been shown to reduce cancer-induced side effects such as nausea, vomiting (Taylor et al., 2020) and peripheral neuropathy (Lynch et al., 2014; Masocha, 2018; Blanton et al., 2019). Several studies in animal models have evaluated the role of the ECS in the modulation of cognitive functions (Schreiner and Dunn, 2012; Cohen and Weinstein, 2018) indicating for example the anxiolytic effects of low doses of cannabinoids. However, only few trials with cannabinoids have evaluated the mood state of cancer patients. ∆-9-tetrahydrocannabinol (THC) and nabilone have been proposed as alleviators for cancer-related psychological disorders, including depression and anxiety (Guzmán, 2003), however they need to be further evaluated though clinical trials. As a matter of fact, to the best of our knowledge no study has ever analysed the potential therapeutic value of cannabinoid drugs in CICI (Kleckner et al., 2019).

In this review we aim to describe the role of the ECS in two well-known CICI-associated processes: neuroinflammation and oxidative stress. In lack of specific studies on the topic, we will review the involvement of the ECS in cancer disease and other pathologies exhibiting similar cognitive phenotype to CICI.

## Cannabinoids and Cancer

From a preclinical perspective, several studies have reported the involvement of the ECS in cancer disease. Increased expression of endocannabinoid receptors and ligand levels have been classically associated with carcinogenesis processes and a higher aggressiveness of cancer (Zhu et al., 2000; Hart et al., 2004; McKallip et al., 2005). Additionally, CB_2_ receptors have been demonstrated to regulate HER2 (human epidermal growth factor receptor 2) oncogene expression, whose upregulation increases vulnerability to leukemia induced by viral infection (Pérez-Gómez et al., 2015).

Regarding the ECS as a therapeutic target against cancer activity, it has been observed its implication in the inhibition of cell proliferation and/or angiogenesis in different tumour types (Śledziński et al., 2020). Attending to cancer cell type and substance, the anti-tumorigenic effects of cannabinoids have been shown to be mediated *via* CB_1_, CB_2_ and TRPV1 receptors. Cell activation of CB_2_ receptors led to a reduced cell motility in bladder cancer, decreasing proliferation rates (Bettiga et al., 2017). The phytocannabinoids THC and Cannabidiol (CBD) have been also reported to exert anti-tumour effects on U-87 MG cell-derived tumour xenografts by decreasing cancer growth *via* cell apoptosis (Torres et al., 2011). THC was shown to induce apoptosis of primary brain tumour cells (Carracedo et al., 2006) and to inhibit tumour growth and survival in a murine Lewis lung adenocarcinoma model (Ramer and Hinz, 2017). Interestingly, knockout mice for CB_1_/CB_2_ receptors exhibited a lower incidence to develop skin cancer after treatment with ultraviolet radioation (Surh et al., 2008). *In vivo* investigations have revealed cannabinoid-inhibition of tumour angiogenesis by inhibition of vascular endothelial cell migration and survival; as well as suppression of proangiogenic factor and matrix metalloprotease (MMP) expression in tumours (Blázquez e al., 2003). Cannabinoid administration has also been associated with a significant decrease in the expression of proangiogenic factors VEGF and Ang2, which result essential for the vascularization of different types of tumours (Carmeliet and Jain, 2000; Casanova et al., 2002). Altogether, the anti-tumour activity, including cancer cell death induction and angiogenesis inhibition, of cannabinoid drugs remark their potential as emergent and effective pharmacological targets in cancer.

Despite the potential anti tumorigenic effects demonstrated in numerous preclinical evaluations only one clinical study tested THC phytocannabinoid as systemically anticancer agent in glioblastoma multiforme (Guzmán et al., 2006). THC was injected intracranially into patients with an early diagnosed glioblastoma. However, the experiment failed to provide strong data supporting THC’s efficacy at that cancer stage. Recent clinical investigations have tested the administration of exocannabinoid compounds, such as Sativex, CBD or dexanabidiol, in different modalities of cancer (e.g. glioblastoma, advanced solid tumours, brain cancer, and neck squamous cell carcinoma); showing reductions in circulating tumour cells, reductions in tumour size, improved survival rate or reduced risk of head and neck squamous cell carcinoma (Moreno et al., 2019). Another possible approach could combine the use of chemotherapeutic agents and cannabinoid drugs to establish whether cannabinoids can enhance the current drug treatments. The few experiments that have investigated this hypothesis have shown controversial results. One study, using γ-radiation combined with a cannabinoid-based treatment demonstrated increased leukemic cell death than single administration of γ-radiation (Jacobsson et al., 2000). However, synergism was not observed when cannabinoids and tamoxifen were combined to induce glioma cell death (Radin, 2003).

It is important to remark that cannabinoids are currently used in palliative medicine for treatment of nausea and vomiting in cancer patients undergoing chemotherapy (Besner et al., 1992; Hall et al., 2001; Walsh et al., 2002). In addition, several preclinical studies have shown beneficial effects of cannabinoid drugs in chemotherapy-induced neuropathy, which is a common side effect of several chemotherapeutic agents, especially platinum-based compounds and taxanes (Abrams and Guzman, 2015; Blanton et al., 2019). Even though the anticancer effectiveness of cannabinoid drugs still remains unclear, its clinical use for the alleviation of cancer side effects such as pain, vomiting, nausea or anorexia is well stablished (Abrams and Guzman, 2015; Dariš et al., 2019; Vecera et al., 2020).

Taking this context into account, the following sections aim to clarify the involvement of the ECS in the two main processes underlying chemotherapy-induced cognitive impairment: neuroinflammation and oxidative stress.

## Cannabinoids and Neuroinflammation

The presence of a tumour and/or the pharmacological management of cancer provokes the activation of the immune system. This mechanism of defence promotes the release of pro-inflammatory mediators responsible for an inflammatory response (Mounier et al., 2020). The pro-inflammatory factors reach the central nervous system (CNS) enhancing the inflammatory response through the activation of glial cells such as microglia and astroglia (Nguyen and Ehrlich, 2020) and promote the release of proinflammatory cytokines such as: tumour necrosis factor alpha (TNFα), interleukin 1 (IL-1) and interleukin 6 (IL-6). A persistent neuroinflammatory response provokes, among others, alterations in neurogenesis and changes in the myelination processes (Mounier et al., 2020), which are responsible for the emergence of cognitive impairments (Fourrier et al., 2019).

The ECS plays a key role in the homeostasis of the immune system. The ECS modulation of the immune system can promote neurogenesis or neurodegeneration (Molina-Holgado and Molina-Holgado, 2010; Tanasescu et al., 2013). Cannabinoid drugs have been used as therapeutic tools in a great number of neuroinflammatory and ageing animal models that involve cognitive dysregulation (Bisogno and di Marzo, 2008; Bilkei-Gorzo, 2012; Chiurchiù et al., 2018; Estrada and Contreras, 2020). As reported below, several studies have analysed the neuroprotective actions of cannabinoid drugs in pathologies that combine neuroinflammatory responses and cognitive impairments, but present different aetiologies, such as Parkinson’s disease (PD), Alzheimer’s disease (AD) or traumatic brain injury (TBI) (Schurman and Lichtman, 2017; Rodrigues et al., 2019; Uddin et al., 2020). In this section, we propose to analyse the modulatory effect of cannabinoids in these neuropathologies to envision the potential beneficial role over CICI.

PD is a progressive and chronic neurodegenerative disorder characterized by the death of dopaminergic neurons in the substantia nigra pars compacta and the presence of intraneuronal inclusions of the protein *a*-synuclein, generally known as Lewy bodies (Braak et al., 2004; Concannon et al., 2015a; Rodrigues et al., 2019). In PD patients and animal models of PD, the ECS is highly dysregulated (Concannon et al., 2015a; Concannon et al., 2015b), suggesting an implication of this system in the pathology and progression of the disease. In addition, it has also been observed that pharmacological modulation of the ECS can induce neuroprotective actions in PD (Aymerich et al., 2018). For instance, the CB_1_ receptor agonist HU-210 exhibited neuroprotective properties to 6-hydroxydopamine (6-OHDA) neurotoxicity *in vitro*. This neuroprotective effect was greater in the presence of glial cells, suggesting that HU-210 neuroprotection depends on its ability to modify this type of cells (Lastres-Becker et al., 2005). Similar results were observed in two animal models of PD-induced neuroinflammation; PD induced by lipopolysaccharide (LPS) and PD induced by 1-methyl-4-phenyl-1,2,3,6-tetrahydropyridine (MPTP). Both animal models exhibited a reduction of microglial activation, thus pro-inflammatory cytokines expression, following HU-210 and WIN55,212-2, CB1 and CB1/CB2 agonists respectively, administration (Chung et al., 2011; Chung et al., 2012). In addition to WIN55,212–2, the CB_2_ receptor agonist JWH155 induced a similar effect against MPTP neurotoxicity, while CB_2_ receptor genetic ablation exacerbated MPTP neurotoxicity (Price et al., 2009). Likewise, CB_2_ receptor knockout mice are more sensitive to the neuroinflammatory effects induced by LPS compared to their wild littermates (García et al., 2011). Additionally, an increase in CB_2_ receptor expression has been positively correlated with an increase of microglial activation (Concannon et al., 2015b) in animal models of neuroinflammation and neutoxocity. Moreover, recent post-mortem studies have shown that there is an increase in the expression of CB_2_ receptors in microglia of the substantia nigra and a decreased expression of this cannabinoid receptor in tyrosine hydroxylase-positive cells in patients suffering from PD (García et al., 2015; Gómez-Gálvez et al., 2016). Additionally, it was detected, in neurotoxic and inflammation-driven animal models of PD, an increase in CB_2_ receptor expression that correlated with an increase of microglial activation (Concannon et al., 2015b), attributing clinical relevance to the involvement of CB_2_ receptors in neuroinflammatory processes associated with PD.

AD is a neuropsychiatric and neurodegenerative disorder with an important neuroinflammatory component. In fact, chronic inflammation contributes to the pathophysiology of AD and is closely associated to the neuropathological and cognitive syndromes of AD (Marchalant et al., 2008; Bonnet and Marchalant, 2015). Several studies have observed that the activation of CB_2_ receptors decrease neuroinflammation in animal models of AD (Uddin et al., 2020). In a recent study the administration of the CB_2_ receptor agonist JWH-015 induced a significant reduction of the gene expression of pro-inflammatory cytokines in the prefrontal cortex of the APP/PS1 double transgenic mice linked to a decrease of the microglial biomarker Iba-1. Yet, CB_2_ activation did not reduce neuroinflammation in the hippocampus or decreased the β-amyloid plaque deposition (Li et al., 2019). In addition, administration of JWH-015 in these transgenic mice improved their working memory in the novel object recognition test, but not their spatial memory measured in the Morris water maze (Li et al., 2019). In the same animal model, the administration of the CB_1_ receptor agonist ACEA decreased astroglial response in the vicinity of β-amyloid plaques and decreased the expression of the pro-inflammatory cytokine interferon-γ in astrocytes (Aso et al., 2012). ACEA also improved the working memory and decreased the activity of Akt and ERK in the hippocampus of another AD animal model consisting in intracerebroventricular administration of streptozotocin (STZ) (Crunfli et al., 2019). CBD is one of the main pharmacologically active phytocannabinoids of the plant *Cannabis sativa L.* (Atalay et al., 2020), but, unlike THC, it does not produce psychotropic effects and presents no affinity to CB_1_ and CB_2_ receptors. *In vitro* studies have described the anti-inflammatory effects of CBD (Esposito et al., 2006a; Esposito et al., 2006b; Martín-Moreno et al., 2011), however, recent *in vivo* studies have failed to relate these effects with a reversion of cognitive impairments in animal models of AD (Cheng et al., 2014a; Cheng et al., 2014b; Watt et al., 2020) which may indicate that CB_1_ and CB_2_ receptors play a crucial role in the cognitive impairments induced by the inflammatory response and they are potential therapeutic targets to take into account in future experiments.

Traumatic Brain injury (TBI) is a non-degenerative disease induced by a mechanical neuronal damage. This type of damage triggers a cascade of neuroinflammatory events usually followed by an increase of endocannabinoid ligand levels: AEA and 2-AG. This effect is thought to be an immediate response to maintain brain-related homeostasis since binding of these ligands to CB_1_ and CB_2_ receptors generate an anti-inflammatory response in an attempt to counteract the injury-related inflammation (Vázquez et al., 2015; Schurman and Lichtman, 2017). AEA levels have been shown to be increased in the brain ipsilateral side of the lesion in different TBI animal models, a compensatory effect that is thought to prevent cell degeneration. Administration of the FAAH inhibitor PF-3845 prevented dendritic loss and restored the levels of synaptophysin, a synaptic transmission precursor, in the ipsilateral dentate gyrus. Furthermore, the administration of PF-3845 (5 mg/kg) reversed TBI-induced impairment of hippocampal-dependent memory. However, since PF3845 not only induced an increase on AEA levels but also 2-AG levels (Tchantchou et al., 2014), both endocannabinoid ligands could be involved in this neuroprotective activity observed in the ipsilateral brain. In addition, CB_1_ receptor antagonists reverted 2-AG anti-inflammatory effects suggesting 2-AG-mediated activation of CB_1_ receptors induce neuroprotection (Panikashvili et al., 2001; Panikashvili et al., 2005; Panikashvili et al., 2006). TBI also induces a significant increase of CB_2_ receptors expression in the injured cortex. Activation of CB_2_ receptors by GP1a (a CB_2_ receptor agonist) induced a significant decrease in the levels of pro-inflammatory cytokines as well as an increase in the number of M2 macrophages in a TBI animal model (Braun et al., 2018). Since CB_1_ and CB_2_ activation plays such an important role counteracting the neuroinflammatory response after TBI it is not surprising that two well-known neuroprotective compounds with no direct relation with the ECS, such as the antibiotic minocycline and the hormone leptin, had their anti-inflammatory properties blocked when CB_1_ and CB_2_ receptor antagonists were administered (Lopez-Rodriguez et al., 2015; Lopez-Rodriguez et al., 2016). Although the use of cannabinoid drugs following TBI has been linked to decreased inflammatory cell activation and decreases in pro-inflammatory cytokine production (Price et al., 2009), little is known about the prevention or reversion of the development of cognitive impairments after TBI.

## Cannabinoids and Oxidative Stress

Chemotherapeutic drugs induce an increase of the mitochondrial production and accumulation of reactive oxygen and nitrogen species (ROS/RNS), a phenomenon known as oxidative stress (Lipina and Hundal, 2016; Atalay et al., 2020; Mounier et al., 2020). Intracellular accumulation of ROS and RNS results in cell damage and subsequent death (Lipina and Hundal, 2016; Umeno et al., 2017; Gallelli et al., 2018; Atalay et al., 2020). Oxidative stress is especially toxic in cancer cells due to their high metabolic rate, however, healthy cells in the CNS can also be damaged by the oxidative stress-related toxicity generated by chemotherapeutic agents (Kawai et al., 2006; Rajamani et al., 2006; Joshi et al., 2007; Tangpong et al., 2007; Liu et al., 2009).

In the past few years, it has been observed a correlation between the ECS and the synthesis of ROS/RNS. For instance, the ECS has been demonstrated to modulate the activity and expression of key enzymes involved in the synthesis of oxygen reactive species in the CNS, such as NOX2 and NOX4 (Lipina and Hundal, 2016; Gallelli et al., 2018). Moreover, AEA is able to partially reverse oxidative stress induced by exposure to hydrogen peroxide in a culture of hippocampal neural HT22 cells. In particular, AEA increased the cellular metabolic rate and decreased the number of apoptotic cells. AEA also increased the expression of the antioxidant enzyme superoxide dismutase (SOD) and decreased mRNA expression of NOX2 provoking a significant reduction of the intracellular levels of ROS. These AEA-related antioxidant effects were attributed to the activation of CB_1_ receptors, since their pharmacological and genetic blockade reversed those effects (Jia et al., 2014). The ECS can also regulate oxidative stress and lipid peroxidation by conveying beneficial free radical scavenging effects or through directly targeting CB_1_ and CB_2_ receptors (Lipina and Hundal, 2016; Gallelli et al., 2018). Interestingly, the beneficial or detrimental effects induced by the activation of cannabinoid receptors on ROS/RNS synthesis, seems to depend on the cell type and the aetiology and stage of the disease, and CB_1_ and CB_2_ receptors seem to have opposite effects in ROS formation. In the murine macrophage cell line RAW264.7, CB_1_ receptor activation promoted ROS formation via phosphorylation of p38-mitogen-activated protein kinase, whereas CB_2_ receptors suppressed this CB_1_ receptor-mediated effect (Han et al., 2009). Interestingly, this opposite action of CB_1_ and CB_2_ receptors has been documented in studies in which the oxidative stress was caused by a chemotherapeutic agent. For instance, acute and chronic administration of doxorubicin increased markers of oxidative/nitrosative stress in the myocardium of CB_1_^+/+^ mice. This effect was attenuated in CB_1_^−/−^ mice, suggesting the implication of CB_1_ receptors in the oxidative stress induced by doxorubicin (Mukhopadhyay et al., 2010a). In addition, CB_1_ receptor agonists, such as AEA and HU-210, increased ROS generation in human cardiomyocytes, and this effect was attenuated by the concomitant application of the CB_1_ receptor antagonists SR1 and AM281 (Mukhopadhyay et al., 2010b). Similarly, cisplatin administration induced a significant increased expression of renal ROS/RNS synthesising enzymes, such as NOX2 and NOX4, and cell death. These deleterious effects were attenuated by the blockade of CB_1_ receptor or activation of CB_2_ receptors thus protecting against tubular damage (Mukhopadhyay et al., 2010a; Mukhopadhyay et al., 2010c; Horváth et al., 2012).

There is a great number of neuropathologies that cause an increment of the oxidative stress, including neurodegenerative diseases that are commonly associated with the development of cognitive deficits such as AD and PD. In fact, the antioxidant properties of cannabinoid drugs and their effect on cognition have been extensively studied in neurodegenerative animal models. In the STZ animal model of AD, a chronic treatment with the CB_1_ receptor agonist ACEA induced a reduction of nitric oxide (NO) accompanied by an improvement of the short- and long-term working memory measured by novel object recognition test (Crunfli et al., 2019). In a neurotoxic animal model of AD, the injection of β-amyloid peptide in the frontal cortex induced an important neural loss in the CA1, CA2 and CA3 hippocampal regions accompanied with the increased expression of biomarkers for apoptosis and gliosis, only 12 days following β-amyloid peptide administration. It was also observed an increase of the pro-oxidative enzyme inducible nitric oxide synthase (iNOS). Acute administration of VDM11, an inhibitor of AEA cellular reuptake, ameliorated the amnesia induced by β-amyloid peptide administration in the passive avoidance task. Interestingly, the significant increase in the hippocampal levels of AEA induced by the repeated administration of VDM11 reduced the neuronal loss and also the expression of iNOS (van der Stelt et al., 2006). A similar effect was observed when CB_1_ receptors were pharmacologically activated by administration of HU-210 or WIN55,212–2 in the MPTP-induced animal model of PD. Treated animals showed enhanced survival of nigrostriatal dopaminergic neurons, suppressed NOX enzymes and decreased ROS production (Chung et al., 2011).

Other cannabinoid-related compounds have recently attracted attention for their neuroprotective and antioxidant properties. One of these compounds is the endogenous lipid mediator PEA. In the 3xTg genetic mouse model, which contains three well established mutations for the development of AD, a chronic treatment for 90 days with ultramicronized PEA resulted in the rescue of the memory deficits typically observed in this phenotype of mice (Scuderi et al., 2018). Interestingly, this treatment also reversed astrogliosis and neuroinflammation, incremented the expression levels of BDNF in the hippocampus and decreased iNOS levels (Bronzuoli et al., 2018). Another different cannabinoid compound that has been extensively studied for its antioxidant properties is CBD. CBD, like other antioxidants, can modify the level and activity of oxidants and antioxidants and interrupt free radical chain reactions (Atalay et al., 2020). CBD administration also reduces the oxidant effects of chemotherapy drugs. For instance, CBD reduced iNOS levels in cardiac tissue and decreased serum levels of NO in mice treated with doxorubicin (Fouad et al., 2013). In addition, in the mouse model of cisplatin-induced nephropathy, CBD markedly attenuated cisplatin-induced oxidative/nitrosative stress, inflammation and cell death, improving renal function (Pan et al., 2009). As previously mentioned, these CBD antioxidant effects are similar to that provoked by the blockade of CB_1_ or the activation of CB_2_ receptors in this same animal model of nephropathy (Mukhopadhyay et al., 2010a; Mukhopadhyay et al., 2010c; Horváth et al., 2012). In the neurotoxic animal model of AD induced by the intracerebroventricular administration of β-amyloid peptide in mice, chronic administration of CBD reduced the hippocampal expression of iNOS and the subsequent NO release (Esposito et al., 2007) and prevented the spatial memory deficits usually observed in this animal model (Martín-Moreno et al., 2011). CBD was also able to recover 6-OHDA-induced dopamine depletion in this animal model of PD, but only when it was administered immediately after the lesion. This effect was accompanied by an increase in the levels of SOD (García-Arencibia et al., 2006).

## Clinical evidence for the use of cannabinoid drugs in cognitive-related diseases

The preclinical findings on the antioxidant and anti-inflamatory effects induced by the pharmacological modulation of the ECS during PD or AD has also been translated into the clinical field. Despite the vast evidence of cannabinoids-induced neuroprotection in TBI, there are still no studies translating those finding into humans. Elevated endocannabinoid levels have been found in the cerebrospinal fluid of PD patients, together with decreased CB_1_ receptors in the basal ganglia (Hurley et al., 2003; Pisani et al., 2005). A small human trial performed in patients suffering from PD revealed that cannabinoid-related drugs such as CBD, nabilone or even cannabis improved motor symptoms an attenuated levodopa-induced dyskinesia. Moreover, resting tremor, rigidity, bradykinesia, and posture were corrected, followed by a decrease on pain sensitivity and amelioriated sleep quality (Carroll et al., 2004; Mesnage et al., 2004; Lotan et al., 2014). CBD has also been associated with diminished REM sleep behavior disorder in PD patiens (Chagas et al., 2014).

A post-mortem study in human brain samples of AD patients showed an increased expression of CB_2_ receptors in microglia associated with β-amyloid-enriched neuritic plaques. This effect was not detected in CB1 receptors expression, suggesting the involvement of CB_2_ dependent mechanisms in this disorder (Benito et al., 2003). Several clincial studies have investigated the effects of dronabinol, which is a synthetic version of THC, in advanced stages of AD. Dronabinol improved side effects associated with late stages of AD such as food intake, sleep duration and circadian rhythm; decreasing also agitation (Volicer et al., 1997; Woodward et al., 2014; Suryadevara et al., 2017).

These reports demonstrate the potential therapeutic activity of cannabinoid drugs to relieve PD and AD symthomatology. However, to the best of our knowledge, there is no clinical evidence of improvement in the cognitive alterations associated with these neurodegenerative disorders yet. Further clinical and preclinical research is required to assess the cognitive-related therapeutic effects that cannabinoid drugs may exert.

## Discussion

**FIGURE 1:**
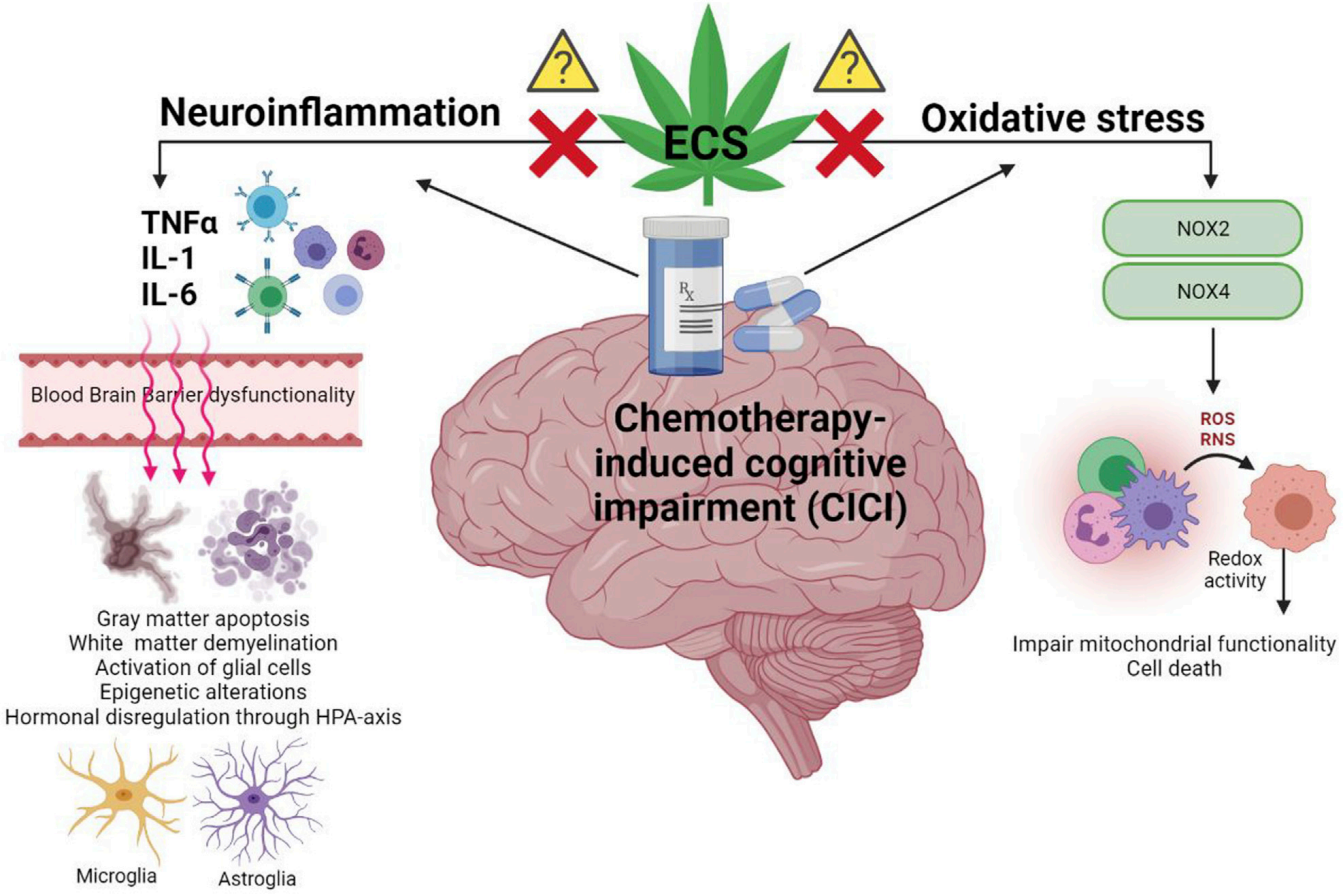
Graphic representation of the neuroinflammatory processes and oxidative stress underlying chemotherapy-induced cognitive impairment (CICI). The activation of the pro-inflammatory cascade, following chemotherapeutic drug administration, is characterized by the central/peripheral release of pro-inflammatory cytokines such as TNFα, IL-1 and IL-6. Transport of these cytokines through the blood brain barrier (BBB) affects its functionality, facilitating, thus, the access of further cytokines and chemotherapeutic drugs into the supraspinal central nervous system (CNS). Therefore, persistent neuroinflammation in the CNS alters brain’s plasticity and functionality mediating the development of cognitive alterations. The commencement of oxidative stress cascade, following chemotherapeutic drug administration, is characterised by the activation of NOX2/NOX4 enzymes which synthesize oxygen and nitrogen reactive species (ROS and RNS, respectively). Increased redox activity due to intracellular accumulation of ROS/RNS induces protein and DNA isoforms alterations that lead to cell death. The endocannabinoid system (ECS), as previously described in similar cognitive-related alterations, proposes a new target for the inhibition of neurotoxicity, providing thus neuroprotection. However, the mechanisms through which the ECS may mediate this process are still unknown in CICI pathology.

Due to the increased survival of cancer patients, there is an urgent need to address the possible sequelae that the current treatments may provoke. Amongst these adverse effects, those affecting cognition and other brain functionality are particularly worrying. The occurrence of chemotherapy-induced cognitive impairment (CICI) has been demonstrated in animal models and human patients. Different biological mechanisms seem to be involved, however there is a big gap in the understanding of those yet. The ECS is implicated in neuroinflammation and oxidative stress (Figure 1). This review comprehends evidence on the use of cannabinoid-related drugs for the modulation of neuroinflammation and oxidative stress in different pathologies with similar cognitive phenotype to CICI, as well as their anti-tumour activity.

The data collected elucidate the positive outcomes of cannabinoid-based drugs in the relief of PD- and AD-side effects in human patients. These results highlight the possible therapeutic potential of cannabinoid drugs in the treatment of CICI. The lack of clinical evidence supporting the anti-cancer role described of the ECS in animal and in vitro models, emphasizes the importance of translating the preclinical findings into humans. This fact points forward the urgent need of clinical assays where the preclinical effectiveness of cannabinoid drugs in the recovery of chemotherapy-induced cognitive alterations can be also investigated.
